# Frequency and severity of myocardial perfusion abnormalities using Tc-99m MIBI SPECT in cardiac syndrome X

**DOI:** 10.1186/1471-2385-6-1

**Published:** 2006-02-17

**Authors:** Mohsen Saghari, Majid Assadi, Mohammad Eftekhari, Mohammad Yaghoubi, Armaghan Fard-Esfahani, Jan-Mohammad Malekzadeh, Babak Fallhi Sichani, Davood Beiki, Abbas Takavar

**Affiliations:** 1 Research Institute for Nuclear Medicine, Tehran University of Medical Sciences, Shariati Hospital, North Kargar Ave. 14114, Tehran, Iran; 2 Department of Cardiology, Shariati Hospital, Faculty of Medicine, Tehran University of Medical Sciences, North Kargar Ave. 14114, Tehran, Iran

## Abstract

**Background:**

Cardiac syndrome X is defined by a typical angina pectoris with normal or near normal (stenosis <40%) coronary angiogram with or without electrocardiogram (ECG) change or atypical angina pectoris with normal or near normal coronary angiogram plus a positive none-invasive test (exercise tolerance test or myocardial perfusion scan) with or without ECG change. Studies with myocardial perfusion imaging on this syndrome have indicated some abnormal perfusion scan. We evaluated the role of myocardial perfusion imaging (MPI) and also the severity and extent of perfusion abnormality using Tc-99m MIBI Single Photon Emission Computed Tomography (SPECT) in these patients.

**Methods:**

The study group consisted of 36 patients with cardiac syndrome X. The semiquantitative perfusion analysis was performed using exercise Tc-99m MIBI SPECT. The MPI results were analyzed by the number, location and severity of perfusion defects.

**Results:**

Abnormal perfusion defects were detected in 13 (36.10%) cases, while the remaining 23 (63.90%) had normal cardiac imaging. Five of 13 (38.4%) abnormal studies showed multiple perfusion defects. The defects were localized in the apex in 3, apical segments in 4, midventricular segments in 12 and basal segments in 6 cases. Fourteen (56%) of all abnormal segments revealed mild, 7(28%) moderate and 4 (16%) severe reduction of tracer uptake. No fixed defects were identified. The vessel territories were approximately the same in all subjects. The Exercise treadmill test (ETT) was positive in 25(69%) and negative in 11(30%) patients. There was no consistent pattern as related to the extent of MPI defects or exercise test results.

**Conclusion:**

Our study suggests that multiple perfusion abnormalities with different levels of severity are common in cardiac syndrome X, with more than 30 % of these patients having at least one abnormal perfusion segment. Our findings suggest that in these patients microvascular angina is probably more common than is generally believed.

## Background

Syndrome X is defined by a typical angina pectoris with normal or near normal (<40% stenosis) coronary angiogram with or without ECG change or atypical angina pectoris with normal or near normal coronary angiogram plus a positive none-invasive test (exercise tolerance test or myocardial perfusion scan) with or without ECG changes [1,2]. Patients with coronary artery spasm (Prinzmetal's or variant angina), left ventricular hypertrophy, systemic hypertension, and valvular heart disease are not included in this syndrome [3]. The term "Microvascular Angina" (MVA) includes all such patients with coronary microcirculatory derangements but with normal coronary angiograms irrespective of the presence or absence of exercise-induced ST segment depression [4].

The exact pathophysiological mechanisms underlying this condition are not well understood, and many mechanisms for the chest pain have been suggested. In some studies, microvascular dysfunction has been proposed as the cause [4-7] whereas in others, metabolic abnormalities, such as net myocardial lactate production have been demonstrated [8-12]. Noninvasive imaging has been used to determine whether ischemia is present or not. Controversial findings have been reported regarding left ventricular function in MVA. Though regional wall motion abnormalities have been reported using stress nuclear techniques, two dimensional echocardiography has not disclosed any segmental contractile dysfunction [13]. Some studies in which positron-emission tomography (PET) was used have shown abnormal heterogeneity in perfusion [14,15] whereas others have shown no abnormality [16,17]. However, the possibility of the development of exercise induced ischemia in these patients is supported by the fact that approximately two-thirds of the patients develop regional abnormalities of left ventricular contraction or show an abnormal ejection fraction response (i.e., less than 5% increment) following exercise [18].

Tl-201 scintigraphy has been employed in the investigation of patients with syndrome X, and often shows regional defects after stress [19-21]. However, the physical limitations due to lower energy emission of Tl-201 as a myocardial perfusion agent have long been recognized [22-25] yielding inconsistent results [19,26]. The development of new and higher energy emission Tc-99m labeled agents, such as Tc-99m MIBI, significantly improved image quality and eliminated false-positives results in Tl-201 SPECT [25,27]. Although, in these patients the diagnosis of microvascular involvement as a "yes-no" binary outcome may be adequate for further management, however, information on both the presence and extent of coronary artery disease (CAD) are desirable, as both have implications in assessment of prognosis or selection of therapy [28-30]. In this study we evaluated myocardial perfusion in syndrome X, using Tc-99m MIBI SPECT.

## Methods

### Participants and study design

The study included 23 females aged 42–58 years (mean 49.86 ± 6.32 [SD] y) and 13 males ranging from 40–59 years (mean, 49 ± 4.61 [SD] y) who had syndrome X. The patients were recruited from the cardiology Clinic at our Hospital from January 2004 to may 2005. This study was approved by the institutional ethics committee of Tehran university of medical science and all patients gave written informed consent.

All patients had a previously established diagnosis of syndrome X, according to the following two criteria; 1- typical history of angina (substernal burning, heavy or squeezing feeling, precipitated by exertion or emotion and promptly relieved by rest or nitroglycerin)[31] and normal coronary angiography (stenosis less than 40%) 2- atypical chest pain (located in the left side of the chest, abdomen, back or arm in the absence of mid-chest sharp, fleeting, recurrent or very prolonged pain unrelated to exercise; not relieved by rest or nitroglycerin but responded to antiacids, or characterized by palpitations without chest pain[31]) with an abnormal exercise electrocardiogram (0.1 mV horizontal or downsloping ST segment depression of 80 msec after the J point) or myocardial perfusion image and completely normal results on coronary angiography, with no inducible spasm on ergonovine-provocation test. In case of the patients with atypical chest pain, other causes especially gastro-esophageal disorders that can mimic this pattern were excluded. None of the patients had diabetes, hypertension, left ventricular hypertrophy (defined as a value above 35 mm for the sum of the heights of the S wave in lead V1 and R wave in lead V5), valvular heart disease, congestive heart failure (CHF), history of myocardial infarction, mitral valve prolapse, left bundle branch block(LBBB), congenital heart disease (CHD), cardiomyopathy, ejection fraction less than 55% in echocardiography or demonstrated any remarkable change in clinical condition during the investigations. Fifteen out of 36 cases had typical whereas other 21 had atypical chest pain. The patients with syndrome X were taking calcium-channel blockers (10), nitrates (8), hormone-replacement therapy (7), beta-blockers (8), potassium-channel openers (5), or no treatment (1 patient). Some of our patients were on more than one medication.

### Myocardial SPECT imaging & analysis

Patients fasted overnight and all cardiovascular drugs were discontinued at least 2 days before the study. All patients were asked to exercise on a treadmill under a standard Bruce protocol. At the achieved peak heart rate (more than 85% the age-predicted maximum heart rate), appearance of typical angina and/or positive exercise ECG findings, 20 mCi Tc-99m MIBI as a compact bolus was injected. The exercise test was considered to be positive if there was a horizontal or downsloping ST segment depression more than 1 mm for 80 microseconds after the J point. An intravenous line of normal saline solution, with a 20-gauge cannula was positioned in an antecubital vein. Imaging was performed 15–30 minutes after exercise. On the next day 60 minutes after injection of 20 mCi Tc-99m MIBI, the patients were asked to eat a fatty meal to accelerate hepatobiliary clearance of Tc-99m MIBI. The resting SPECT was performed 90 minutes after Tc-99m MIBI injection. SPECT images were obtained using a double detector system (ADAC Genesys Malpitas, CA, USA) with low-energy, all purpose (LEAP) collimator. For Tc-99m MIBI SPECT, a symmetric 15% window was centered at 140 KeV, and images were acquired into a 64 × 64 computer matrix through a 180° rotation, at an angular interval of 6° from RAO 45° to LPO 45°. Reconstruction was performed by a standard back projection method using a Butterworth Filter for a 64 × 64 matrix image. Acquisition parameters were identical for the rest and stress studies.

**Figure 1:**
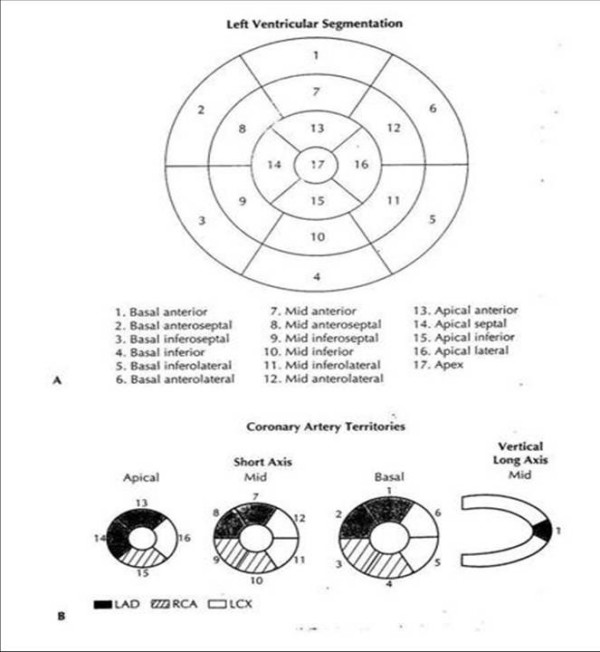
A. A polar-plot depiction of the left ventricular segmentation according to the 17-segment model. The recommended nomenclature is noted for each segment below. B. The 17-segment model, obtained by three individual short-axis slices as well as one midcavity vertical long-axis slice. A depiction of the coronary artery distribution is also noted.

Interpretation was made on the basis of a left ventricular polar-plot segmentation according to the 17-segment model [32] [Fig 1]. This was done by two observers who had no previous knowledge of the patients' histories, results of exercise ECG, or coronary angiograms. The uptake in each of these segments was assessed as normal, mild (= equivocal), moderate, severe or absent [33].

### Coronary arteriography

Coronary arteriography was performed with a monoplane imaging system and recorded on conventional 35 mm film. Angiographic images were visually assessed by two readers who were blind to MPI data.

### Statistical analysis

The statistical analysis was performed with the use of SPSS version 11.5. Data are presented as the mean ± one standard deviation. Chi square test was also applied. A p value < 0.05 was considered to be statistically significant.

## Results

**Figure 2:**
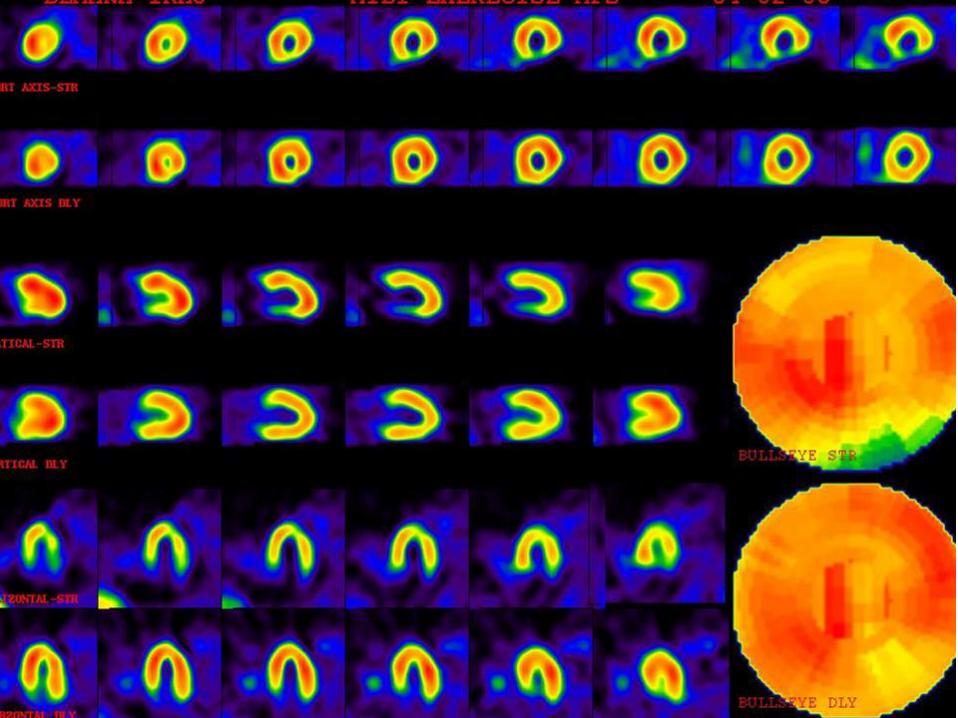
A patient with multiple perfusion defects with different degrees of severity. The upper images are stress phase and lower images are rest or delayed images. There is moderate ischemia in the mid anterolateral and mid inferolateral walls. Also there is severe ischemia in the basal inferior wall. Mild ischemia in the apex, apical septal and basal anterolateral are also noted.

Thirteen patients out of 36 (36.1%) showed abnormal myocardial perfusion scan. Of the 23 females patients, 9 (39.1%) had abnormal Tc-99m MIBI SPECT and 14 (60.9%) showed normal scan. Of the 13 males 4 (30.8%) showed abnormal and 9(69.2%) cases revealed normal studies. There was no significant difference between two genders (p value > 0.05). Of the 9 positive female patients, 3 (33.33%) cases showed multiple perfusion defects and 6(66.66%) cases revealed single localized perfusion abnormality. Of the 4 males with positive scan 2 (50%) cases revealed multiple and 2 (50%) single perfusion defects. The defects were localized in the apex in 3, the apical segments in 4, the midventricular segments in 12 and in the basal segments in 6 cases. All patients had reversible perfusion abnormality and none of them had transient left ventricular dilation (TLV). The involvement of three vessel territories approximately were the same(LAD;8, RCA;9 and LCX;8). In the cases with multiple perfusion defects, the ischemic pattern was distributed throughout the different segment of myocardium. The detailed data of uptake reduction are shown in table 1 and a sample picture is presented [Fig. 2]. Exercise treadmill test (ETT) was performed in this study as a part of the protocol. The results of ETT were positive in 25 (69%) patients and negative in 11(30%). 65% of the results of the exercise ECG were not concordant with perfusion defects found on myocardial perfusion imaging.

**Table 1:** frequency and percent of MIBI uptake defects and involved segments of coronary vessel territories

	LAD		LCX		RCA		Total	
	
	No	%	No	%	No	%	No	%
Mild	6	75	3	37.5	5	55.5	14	56
Moderate	1	12.5	4	50	2	22.2	7	28
Severe	1	12.5	1	12.5	2	22.2	4	16
Total	8	100	8	100	9	100	25	100

Abbreviation: LAD = Left anterior descending artery, LCX = Left circumflex artery, Rca = Right coronary artery

## Discussion

Investigations over the past two decades have not revealed a specific cause for syndrome X. In the presence of normal coronary arteries, many explanations have been put forth to explain myocardial ischemia, including small vessel abnormalities, coronary artery spasm, cardiomyopathy, metabolic abnormalities, misinterpretation of the coronary angiograms, impaired coronary flow reserve, oxyhemoglobin dissociation defects, psychosomatic factors, altered pain perception, increased sympathetic drive, and endothelial dysfunction [33,34]. It is now acknowledged that syndrome X most likely encompasses several pathophysiologic diseases. Since the introduction of the term syndrome X, many investigators have used the term with different criteria as to its definition, to create a more homogeneous group of patients. Our results suggest that multiple perfusion defects with different severity and distribution are relatively common in cardiac syndrome X, with the majority of these patients having at least one abnormal perfusion segment. Thus, 36 % of our patients with angina pectoris, who had no evidence of significant organic stenosis on their coronary angiograms, exhibited exercise-induced perfusion defects in their Tc 99m-MIBI scintigrams. In a review of the literature, a number of authors have reported different results when using conventional Tl-201 myocardial perfusion imaging. Berger et al reviewed the exercise Tl-201 scans and clinical data of 41 patients with chest pain and normal coronary arteries (a broad definition of syndrome X). A negative Tl-201 study was the most common finding. Of the 41 patients, 11 (27%) had abnormal exercise Tl-201 scans. Of the 11 patients with abnormal scans, 9 had greater than or equal to 1 cardiac abnormality [19]. Tweddel et al (1992) studied 100 patients with normal arteriograms (a broad definition of syndrome X) undergoing diagnostic angiography for typical angina. Thallium defects were found in 98 patients (a very high incidence) [20]. Legrand et al (1985) studied the results of exercise Tl-201 scintigraphy in 18 patients with chest pain and angiographically normal coronary arteries (a broad definition of syndrome X). Regional exercise Tl-201 perfusion was abnormal in three patients [21]. Chia-Hung et al (1996) reviewed the stress Tc-99m MIBI SPECT results and clinical data of 15 patients with syndrome X. The results of exercise ECG and resting left ventricular ejection fraction (LVEF) were compared with the results of Tc-99m MIBI SPECT. Of these 15 patients, 9 (60%) had a normal Tc-99m MIBI SPECT study and 6 (40%) had an abnormal Tc-99m MIBI SPECT [35]. Recently Cavusoglu et al (2005) have evaluated the regional distribution and extent of perfusion abnormalities, and the lung to heart (L: H) uptake ratios using exercise thallium-201 SPECT in 31 patients. They showed that perfusion abnormalities are relatively common in these patients. Also patients with perfusion abnormalities have significantly higher L: H ratios during exercise than control patients [36]. Jonathan et al (2002) performed myocardial-perfusion cardiovascular magnetic resonance imaging in 20 patients with syndrome X and 10 matched controls, both at rest and during an infusion of adenosine. Quantitative perfusion analysis was performed using the normalized upslope of myocardial signal enhancement to derive the myocardial perfusion index and the myocardial-perfusion reserve index (defined as the ratio of the myocardial perfusion index during stress to that at rest). Following adenosine, control group revealed increased myocardial perfusion in subendocardial and subepicardial layers of myocardium but there was only increased perfusion in subepicardium in patients with syndrome X without any detectable change in the subendocardial layer [37]. In our study, 69 % of exercise tests were abnormal which is similar to the study by Cannon et al (1985) [18]. In this study, we tried to match coronary anatomy with perfusion segments to localize CAD to the level of small vessels, its potential application being in serial studies, following surgical or percutaneous revascularization interventions, and in patients without an established diagnosis of CAD to identify small sized ischemia or infarctions, not detectable by angiography. Our study suggests that SPECT MPI can be used to identify small perfusion defects pointing to the presence of CAD and also suggests that this procedure may have value in the assessment of the physiological significance of small vessel stenosis not identifiable by coronary angiography. We noticed that the ischemic regions in patients with multiple perfusion defects are distributed in the different myocardial segments as previously reported by other investigators [38].

Furthermore, ischemia may not necessarily be localized to an entire myocardial area as in patients with epicardial coronary stenosis.

Indeed myocardial ischemia may be patchily distributed in small areas of myocardium. In these patients, small areas of hypoperfusion were patchily distributed in different anatomic sites, spreading among nonischemic areas, able to compensate for the dysfunction of adjacent myocardium. This pattern is clearly different from that of patients with coronary artery disease, whereas the hypoperfused areas are clustered in the anatomic regions supplied by the stenotic arteries [38].

In addition, in theses patients inappropriate constriction of prearteriolar vessels may be postulated to be sparse in the myocardium and to be non uniform with the possibility that only a minority of these vessels are intensely constricted. Thus, during increased myocardial activity a focal and spotty myocardial ischemia can result although electrocardiographic signs, metabolism and function are found to be within normal limits. Similarly, a focal increase in myocardial adenosine concentration may cause anginal pain even in the absence of significant myocardial ischemia. Depending on the percentage of involvement of prearteriolar abnormality reduced coronary flow reserve and the presence of detectable myocardial ischemia by nuclear scan may vary widely [39].

The patients in our study had well-characterized syndrome X; however, other conditions may lead to microvascular dysfunction, and similar findings might be found in patients with hypertension, hypertrophic conditions, CHD, CHF, LBBB, MVP, valvular diseases, cardiomyopathy or diabetes. Therefore, we excluded patients with these conditions from our study. Although there is predominance of female to male patients (23/13) referred to our hospital, the scan abnormalities are almost similar in both genders (39% Vs 38%). The prognostic implications of the identification of microvessel disease are uncertain. Although one may expect that, if the total myocardium at risk is small, the patient's prognosis will be favorable, the significance of CAD in multiple branches has not been assessed. Our study did not address the prognostic implications of branch vessel disease, as detected by SPECT MPI. Further studies are needed to follow these patients and determine the prognostic significance of these interesting findings.

Finally it should be emphasized that our study is not free of drawbacks. Since the sensitivity of SPECT as compared to PET for the assessment of myocardial ischemia is less than optimal, the absence of documented myocardial ischemia does not allow for the exclusion with certainty of cardiac and/or ischemic genesis of anginal pain in those remaining patients, in whom nuclear scan was normal [40].

## Conclusion

Our results support the concept that the chest pain in patients with syndrome X may be related to myocardial ischemia. Myocardial perfusion defects revealed by scintigraphy in 13 out of 36 (36 %) patients with angina and normal coronary arteriograms, raises the possibility of microvascular angina as the cause of chest pain. The term "microvascular angina" is commonly used in these patients; however, the cardiologists should be cautioned when confronting cases with discordant scintigraphic and angiographic findings.

## Competing interests

The author(s) declare that they have no competing interests.

## Authors' contributions

MS participated in the interpretation of the scintigraphic results. MA participated in its design and coordination, supervised the acquisition process, participated in the interpretation of the scintigraphic results and also participated in writing of this manuscript. ME supervised the acquisition process, interpreted the scintigraphic results and edited the manuscript. MY carried out angiography and interpreted exercise test. AFE supervised the acquisition process. JMM performed the statistical analysis. BFS, DB and AT supervised the acquisition process. All authors read and approved the final manuscript.

## Pre-publication history

The pre-publication history for this paper can be accessed here:


